# Coupling and de-coupling of the El Niño Southern Oscillation to the supply of larval fishes to benthic populations in the Hawaiian Islands

**DOI:** 10.1371/journal.pone.0312593

**Published:** 2024-10-24

**Authors:** David B. Carlon, S. Maria Garcia, Anuschka Faucci

**Affiliations:** 1 Department of Biology, University of Hawaii at Manoa, Honolulu, Hawaii, United States of America; 2 Department of Biology, Bowdoin College, Brunswick, Maine, United States of America; Xi’an Jiaotong University, CHINA

## Abstract

Several recent high intensity ENSO events have caused strong negative impacts on the adult phases of foundational species in coral reef ecosystems, but comparatively little is known about how climatic variables related to recent ENSOs are impacting the supply of larvae to benthic populations. In marine fishes and invertebrates, reproductive adults and planktonic larvae are generally more sensitive to environmental variability than older, non-reproductive adults. Further, the transport of larvae in ocean currents may also be strongly ENSO dependent. The interactions between the dynamics of larval survivorship and larval transport could lead to population bottlenecks as stronger ENSO events become more common. We tested the predictions of this hypothesis around the Main Hawaiian Islands (MHI) by constructing a correlation matrix of physical and biological time series variables that spanned 11 years (2007–2017) and multiple ENSO events. Our correlation matrix included four types of variables: i. published ENSO indices, ii. satellite-derived sea surface temperature (SST) and chlorophyll variables, iii. abundance and diversity of larval fishes sampled during the late winter spawning season off Oahu, and iv. abundance and diversity of coral reef fish recruits sampled on the western shore of the Big Island of Hawaii. We found that the abundance and diversity of larval fishes was negatively correlated with the Multivariate El Niño Index (MEI), and that larval variables were positively correlated with measures of fall recruitment (September & November), but not correlated with spring-summer recruitment (May & July). In the MHI, SST variables were not correlated with the MEI, but two successive El Niño events of 2014–15 and 2015–2016 were characterized by SST maxima approaching 30°C. Two large pulses of benthic recruitment occurred in the 2009 and 2014 recruitment seasons, with > 8000 recruits observed by divers over the summer and fall months. Both events were characterized by either neutral or negative MEI indices measured during the preceding winter months. These patterns suggest that La Niña and the neutral phases of the ENSO cycle are generally favorable for adult reproduction and larval development in the spring and summer, while El Niño phases may limit recruitment in the late summer and fall. We hypothesize that episodic recruitment during non-El Niño phases is related to favorable survivorship and transport dynamics that are associated with the formation of pairs of anticyclonic and cyclonic eddies on the leeward sides (western shores) of the Main Hawaiian Islands.

## Introduction

There are growing concerns that anthropogenic climate change is amplifying climate oscillations and their impacts on ecosystems. In the tropical Pacific Ocean, the El Niño—Southern Oscillation (ENSO) is a key climate oscillation that alternates between a warm “El Niño” phase and a cooler “La Niña” phase with quasiperiodic frequency on time scales of two to seven years. The warm, “El Niño” phase is forced by weakening trade-winds across the Pacific Ocean which causes warm ocean surface water to flow eastward towards the Americas. During “El Niño” the sea surface temperature (SST) across the Central and Eastern Pacific increases, and the thermocline deepens. Intensification of the trade-winds reverses this trend, and when trade-winds are stronger than usual, warm water is pushed across the Pacific towards the Indo-Pacific, starting the La Niña cycle. Consequently, SST is cooler in the Central and Eastern Pacific, the thermocline decreases in depth, and upwelling along the coast of the Americas intensifies. Through these changes in physical oceanography, ENSO can precipitate dramatic changes in ecosystem function. An iconic El Niño impact on marine ecosystems results from the reduction in upwelling and its associated productivity along the coastal margins of the eastern Pacific which has bottom-up effects on the phytoplankton communities and their secondary consumers [1]. The El Niño phase of the ENSO cycle is also amplifying anthropogenic increases in sea water temperature over the last century, pushing narrowly adapted tropical marine species [2] closer to their upper thermal tolerances. This ENSO effect on tropical ecosystems is illuminated by the particularly intense El Niño of 2015–2016 which brought exceptionally high sea surface temperatures (SSTs) to the Great Barrier Reef system for several weeks. The result was widespread coral bleaching and coral mortality over 1000s of kms of reef tract [3–5]. The ENSO cycle can also have strong effects on subtropical estuarine fish communities, either through salinity impacts on euryhaline fishes [6] or through temperature mediated changes in the abundance of keystone seagrass species [7]. Given that the frequency of extreme ENSO events appears to be increasing during this last century [8] and even in the last few decades [9], there is an urgency to understand how these climate oscillations impact the structure and function of a diversity of subtropical and tropical marine ecosystems.

The impacts of ENSO include fundamental changes in the physical and biological oceanography of surface waters, which raises the possibility that planktonic organisms and larvae are facing new physiological challenges. The life cycle of many marine species includes a larval phase that feed and develop in the upper mixed layer of the ocean; and may take days, weeks, or even months to complete larval development. In tropical reef fishes, for example, the planktonic larval duration can range from 12–39 days in the damselfishes, but as long as 131 days in the wrasses [10]. The elevated sensitivity of these early life history phases to changes in the environment is supported by a recent meta-analysis of the world’s fishes that has shown spawning adults and early life-history phases have much narrower tolerances to temperature stress compared to non-reproductive adults [11]. The reduced performance of early life history phases at the upper margins of thermal tolerance is hypothesized to be driven by a more limited ventilation and oxygen transport capacity in small embryos and larvae [12, 13]. Consequently, the aerobic scope for maintenance and growth of larvae may decline more rapidly with temperature in contrast to adult phases. A second challenge to maintaining physiological homeostasis in feeding larvae in the face of rising sea surface temperatures are the additive effects between temperature stress and a reduced or unpredictable food supply [14]. Experimental studies in larvae of temperate and tropical fish species have demonstrated that reducing the food supply at temperatures near thermal maxima decreases the scope for growth [15, 16], or the number of mating pairs in reproductive adults [17]. From an energetic standpoint, the warmer temperatures and lower productivity associated with the El Niño phase of ENSO may present considerable challenges to feeding planktonic larvae. Consistent with a “metabolic meltdown” of reproduction during stressful ENSO events, a longitudinal study has linked reduced ocean productivity and declines in the supply of larval fishes to the very strong 1997–1998 El Niño event on Rangiroa Atoll, French Polynesia [18]. This previous body of physiological and ecological studies suggest that high intensity ENSO events could significantly decrease larval survivorship at this critical stage, leading to larval bottlenecks in many tropical marine populations.

The problem of understanding how ENSO impacts the larval side of benthic population dynamics depends on how changes in water column properties and processes (e.g. temperature, food supply, consumption rates by predators) that impact the survivorship of larvae are coupled with changes in the structure of ocean currents that transport these developing larvae back to shore. Thus, a second dimension of ENSO-related drivers of marine population dynamics is the impact of changes in physical oceanography on the transport of larvae back to adult habitats. Several studies have now linked ENSO driven changes in the speed and direction of coastal currents to the dispersal trajectories of developing larvae [19–22]. On the Great Barrier Reef (GBR), an eastward flowing Southern Equatorial Current bifurcates in the middle of the GBR system with one branch flowing northward and a second branch flowing southward. Whether one branch dominates this flow pattern depends on ENSO phase, and Gurdek-Bas et al. [22] have used a biophysical model to show that the net northern or southern dispersal of snapper larvae will largely depend on ENSO phase. These examples suggest that ENSO-forced changes in surface layers can impact both the ecological performance and transport of larvae back to suitable nearshore habitats.

The Hawaiian Islands are a particularly tractable system to study the linkages between ENSO, larval dynamics, and benthic recruitment. Extending across the margins of the tropics (19° N, Big Island of Hawaii– 28° N, Kure Atoll), the physical and biological oceanography around the Hawaiian Islands are relatively well studied. The existing time series of coral reef fish recruitment on the Big Island of Hawaii known as the West Hawaii Aquarium Project (WHAP, http://www.coralreefnetwork.com/kona/, [23, 24]) provides an opportunity to study the potential linkages between key water column processes forced by ENSO, larval dynamics in the plankton, and subsequent recruitment dynamics on the benthos (e.g. [25]). In this study, we focus our analysis on a suite of water column variables, new measurements of the planktonic community of fish larvae, and the WHAP recruitment data from the Main Hawaiian Islands (MHI) collected over 11 years, from 2007 to 2017. This time frame captures three El Niño and three La Niña events, including an intense event known as the “Godzilla” El Niño of 2015–2016. We use a correlation matrix to estimate the sign and significance of linkage among these variables, and to identify potential causal factors that may be ultimately driving planktonic and benthic population dynamics. To fully characterize the dynamics of diversity in Hawaiian ichthyoplankton, we present a unique time series of larval fishes sampled of the coast of Oahu, that has been taxonomically identified by both expert visual identification and larval DNA barcoding.

## Methods

### Timing of sampling—Planktonic larvae and recruits

Spawning by reef fishes of the Main Hawaiian Islands (MHI) can occur all year round depending on the species, but the number of species that are reproductively active begins to increase in February when water temperatures are at seasonal lows, and steadily increases until peaking in June–July and before temperatures reach a seasonal maximum during a September–October window [26]. The recruitment of reef fishes to adult coral reef habitat historically occurs in two peaks, starting with a small increase in recruitment in February followed by a major recruitment pulse that can occur between June and August. Recruitment of reef fishes on the Big Island of Hawaii tends to be dominated by two common species: the yellow tang *Zebrasoma flavescens* and the goldring surgeonfish *Ctenochaetus strigosus* [27], but may include up to 49 species in any sampling month (see Results). Our larval sampling was limited to January and February, and therefore does not represent the entire spawning season in Hawaii. On the other hand, we sampled a diversity of reef fishes, offshore species, and deep-sea species. The surveys of benthic recruits were conducted in May, July, September, and October of each year, and are therefore representative of the entire spawning season. Given a planktonic larval duration of a few months, we expected the strongest coupling between larval supply and recruitment to occur in the early spring. For example, fish recruiting in May are likely to originate in spawning events that occurred in the preceding February or March depending on distribution of planktonic larval durations for the recruiting species.

### Larval sampling and DNA barcoding

We conducted annual sampling of the Hawaiian fish larvae with a sets of oblique plankton tows made from day cruises on a small coastal oceanography vessel. These cruises were part of a laboratory exercise for the University of Hawaii undergraduate course “Biol 301L Marine Ecology and Evolution.” Students participated in the collection, sorting, and DNA sequencing of all samples. Cruises and tows were made from a station located west of the city of Honolulu, Oahu (21°15’34", -157°51’00") and began in either January, February, or early March depending on the sampling year (S1 Table). Annual samples were pooled from multiple cruise days within a single week, with the number of cruise days determined by enrollment and the number of lab sections that year (range 2–4). On each cruise day we used a 500-micron mesh plankton net with a 0.5 m hoop diameter and weighted with a hoop depressor to make a single oblique tow at each of three depths: 1 m, 15 m, and 25 m. We used a rapid deployment until the target depth was reached, followed by rapid retrieval of the net at the end of the tow. The three specific depths (*d*) were sampled by maintaining a cable angle of 45 degrees with ship speed and deploying the towing cable at *x* length, where *x* = *d*/sin(45°). Tows were made by maintaining a ship course parallel to the 180–200 m depth contour, which occurs between 1–2 km west of the city of Honolulu. For all years, the average tow duration was 18.6 minutes (range: 10–44 minutes). Generally, one set of three depth-specific tows was made during each cruise day, but for 2013 and 2014 two sets of the three specific depths were made on a few of the cruise days. A mechanical flow-meter (General Oceanics, Mechanical Flowmeter, 2030R, Miami, FL) was used to measure the tow volume and normalize sampling effort starting in 2012, but we did not reliably collect flowmeter data for the previous years. To normalize all our larval counts by sampling effort, we used the total number of tows made during all cruises for each year. Since tows were always conducted in sets of three-depths, each depth was sampled representatively regardless of the number of cruises made each year. We realize the number of tows per year is a courser measure of sampling effort than total volume of water sampled per year, but for the years and tows in which we had flowmeter data, there was a strong positive relationship between the number of tows and the total volume of water sampled (r^2^ = 0.72, p = 0.015). To control for differences in sampling effort among years on measures of larval diversity (number of species and families) we used a partial correlation approach with the number of tows included as a covariate because the relationship between sampling effort and diversity is typically non-linear in tropical systems. See the **Analyses** section below for details. We did not conduct any depth-specific analysis in this paper. Most taxa were sampled across all depths, but a few species were more common at 25 m, these include species in the Gobiidae, Molidae, and the Myctophidae. We refer the interested reader to the tables of S6 and S7 Tables which report the larval counts by taxon and depth.

After retrieving the net, all zooplankton were thoroughly rinsed into the cod end with seawater. Each tow sample was split with a Folsom plankton splitter, and ½ of the sample was fixed by adding an equal volume of 95% EtOH. To reduce the suffering of larval fishes placed in EtOH, the entire sample was chilled on ice for 1 hour before adding EtOH. The fixation in EtOH rapidly euthanized larval fishes. Samples were transported to a teaching laboratory on the UH Manoa campus on the evening of each cruise, where students sorted all fish embryos and larvae from the fixed samples under instructor supervision. Fish larvae were also sorted from the non-fixed ½ of the tow samples by the lab instructor. After sorting the fixed and non-fixed portion of the sample for each tow, each fish larva was identified to the lowest taxonomic level possible by Bruce Mundy, an expert in tropical larval fishes, and now retired from NOAA’s Pacific Island Fisheries Science Center. The key of Miller et al. [28] was consulted for identifications. After morphological identification, individual eggs, embryos, or larvae were placed in a labeled microcentrifuge tube that was prefilled with 95% EtOH for downstream DNA extraction. Challenges to characterizing the diversity of larval fishes in the plankton with visual identification include the inability to identify the earliest larvae phases to species due to a lack of morphological characters, the inability to use morphology to separate closely related species, and the fact that some samples may be damaged with bulk sampling methods such as plankton nets [29]. DNA barcoding offers an alternative to these identification challenges by offering the prospect of species-level identification based on a reference library of DNA sequences [30]. The technique has recently been applied to samples of larval fishes in several coral reef systems [31, 32], where > 70% of the sampled larvae were identified to the species level. In addition to expert visual identification, we therefore incorporated a DNA barcoding protocol. Students extracted DNA and sequenced the mitochondrial cytochrome oxidase 1 gene (*mtCO1*) in a representative subset of the total larval sample. Genomic DNA was isolated from a small tissue sample removed from the larger larvae, or in the cases of eggs, embryos, or smaller larvae the entire specimen was used. DNA was extracted from tissues with the Qiagen DNeasy kit (Valencia, CA) following the manufacturer’s instructions. We amplified a portion of *mtCO1* gene using the FishF1 and FishR1 primers and the PCR profile described in [30]. The PCR products were prepared for sequencing using an enzymatic incubation in exonuclease and shrimp alkaline phosphatase, then sequenced using the ABI 3700 platform in the ASGBP core facility at the University of Hawaii. Sequences were trimmed and edited using Geneious software (Biomatters, Auckland, New Zealand), and uploaded to a Barcode of Life Data System (BOLD) as a project named FLHI. We ran the BOLD ID engine on the entire FLHI project which consisted of 1575 sequences on May 6, 2021, and used ≥ 98% sequence identity for species identification, and ≥ 80–98% sequence identity for families. Matches of < 80% sequence identity were considered unidentifiable by the current BOLD database.

The collection of animals used in this study did not require permits from the Hawaii State Department of Aquatic Resources and complied with US Animal Welfare Act laws and the guidelines and policies as approved by University of Hawaii Institutional Animal Care and Use Committee which determined that no animal use protocol was required.

### Benthic recruitment

Data on benthic recruitment of reef fishes were collected by the West Hawaii Aquarium Project (WHAP) and provided by Chris Teague (State of Hawaii, Division of Aquatic Resources). The WHAP network consists of 23 sites on the Kohala–Kona-Ka’u coast on the western side of the Big Island of Hawaii (see [25] for site locations). For each census, both juvenile and adult fishes were visually counted along four transects (100 m^2^ each) by trained divers on SCUBA. A fish was counted as a recruit if its estimated standard length was less than a species-specific size threshold for a fish less than one year in age. We used the recruit data for all species summed over transects and sites to calculate the total recruitment for each sampling month, and total annual recruitment by summing total recruitment for each month/year. To determine if only a few species were dominating years with exceptionally high recruitment, diversity was also estimated from the recruitment data, and was calculated as the total number of sampled species/month/year.

### Physical and biological variables related to ENSO

To test for associations between larval supply variables and ENSO forced water column variables, we extracted a set of physical and biological variables from online sources. For a Pacific-Wide measure of ENSO intensity, we used the Multivariate El Niño/Southern Oscillation index (“MEI v2”, downloaded from: https://psl.noaa.gov/enso/mei/) and El Niño 3.4 index (https://www.ncdc.noaa.gov/teleconnections/enso/sst). The MEI is a multivariate score representing five different ENSO variables: sea level pressure, sea surface temperature, zonal and meridional components of the surface wind, and outgoing longwave radiation; over the tropical Pacific basin, from 30°S-30°N and 100°E-70°W. The El Niño 3.4 index measures the sea surface temperature (SST) anomaly in the Central Pacific, from 5N-5S and 120-170W. Since the ENSO cycle typically begins in the boreal winter months, we used the January values of the MEI, El Niño 3.4 anomaly, and El Niño 3.4 sea surface temperature in our downstream correlation matrix. To characterize the water column around the Hawaiian Islands during our plankton sampling time series, we extracted high resolution temperature and temperature anomaly data from NASA JPL Dataset ID: jplMURSST41; and chlorophyll-*a* (Chl-*a*) data from Dataset ID: erdMH1chlamday. We used the R packages rerddap (v1.0.2; Chamberlain 2023, _rerddap: General Purpose Client for ’ERDDAP’ Servers_ <https://CRAN.R-project.org/package=rerddap>) and rerddapXtracto (v1.1.4, Mendelssohn 2022, _rerddapXtracto: Extracts Environmental Data from ’ERDDAP’ Web Services_ <https://CRAN.R-project.org/package=rerddapXtracto>) for downloading and manipulating these satellite derived data sets. Our data grid was bounded by latitude: 19°N—22.5°N and longitude 154.5°W—160°W and included all the Main Hawaiian Islands (MHI), from Kauai in the North to the Big Island of Hawaii in the south. We calculated daily means across this grid of sea surface temperature (SST), SST anomaly, and Chl-*a* concentration. From these daily means we then calculated annual means, minimums, and maximums, for the downstream correlation matrix. In addition, to represent average sea surface conditions during the winter months, we calculated the average SST and Chl-*a* biomass for the six months prior to larval sampling.

### Analyses

To test for correlations among all variables we calculated Pearson correlation coefficients and determined their statistical significance by bootstrapping using the confintr R package (v1.0. 2; Mayer 2023, _confintr: Confidence Intervals_<https://CRAN.R-project.org/package=confintr>). Our bootstrapping approach was more suitable for these data given the assumption of bivariate normality required for parametric approaches to hypothesis testing. We considered a co-efficient as significant by bootstrapping if the 95% confidence intervals did not contain 0. To visualize patterns by magnitude and sign of all correlations in the matrix, we grouped variables according to three categories: i. “Larval supply”—variables derived from the larval sampling and barcoding time series, ii. “Benthic recruitment”—variables derived from the WHAP survey data, and iii. “Sea surface”—variables derived from the extraction of satellite derived temperature and chlorophyll data or published ENSO indices. We used the corrplot R package (v 0.92; Wei and Simko 2021, _R package ’corrplot’: Visualization of a correlation matrix_. <https://github.com/taiyun/corrplot>) to graph the correlation matrix as a heat map.

To determine if the negative relationship we detected between ENSO and the supply of larval fishes also held for fishes that are shallow reef specialists we filtered the larger data set for those larvae that were identified to families that specialize on coral reefs to create the variable “Reef_fish_larval_abundance.” For a list of the coral reef fish families included in this group, see S3 and S7 Tables. Similarly, to test the same relationship for mesopelagic specialists, we filtered the larger data set for lanternfishes (Myctophidae) to create the variable “Myctophidae_larval_abundance.” Larvae from the Myctophidae were the most common samples representing meso-pelagic fishes in the larger data set. Each of these variables were also normalized by tows/year for the correlation analysis.

In contrast to the linear relationship expected for the number of larvae in each tow and sampling effort, measures of larval diversity are expected to saturate at higher sampling effort as the maximum diversity in the environment is approached. To control for this effect on larval diversity estimates we used partial correlation models that included the number of tows made each year as a covariate. We used the ppcor R package (v 1.1; Kim 2015, _ppcor: Partial and Semi-Partial (Part) Correlation_. <https://CRAN.R-project.org/package=ppcor>) to calculate partial correlation coefficients and their significance.

A list of all variables used in the correlation matrix and their definitions can be found in S2 Table. All analyses and graphics were performed using R Statistical Software (v4.3.0; R Core Team, 2023-04-21).

## Results

### Time series and correlation analysis

**Fig 1:**
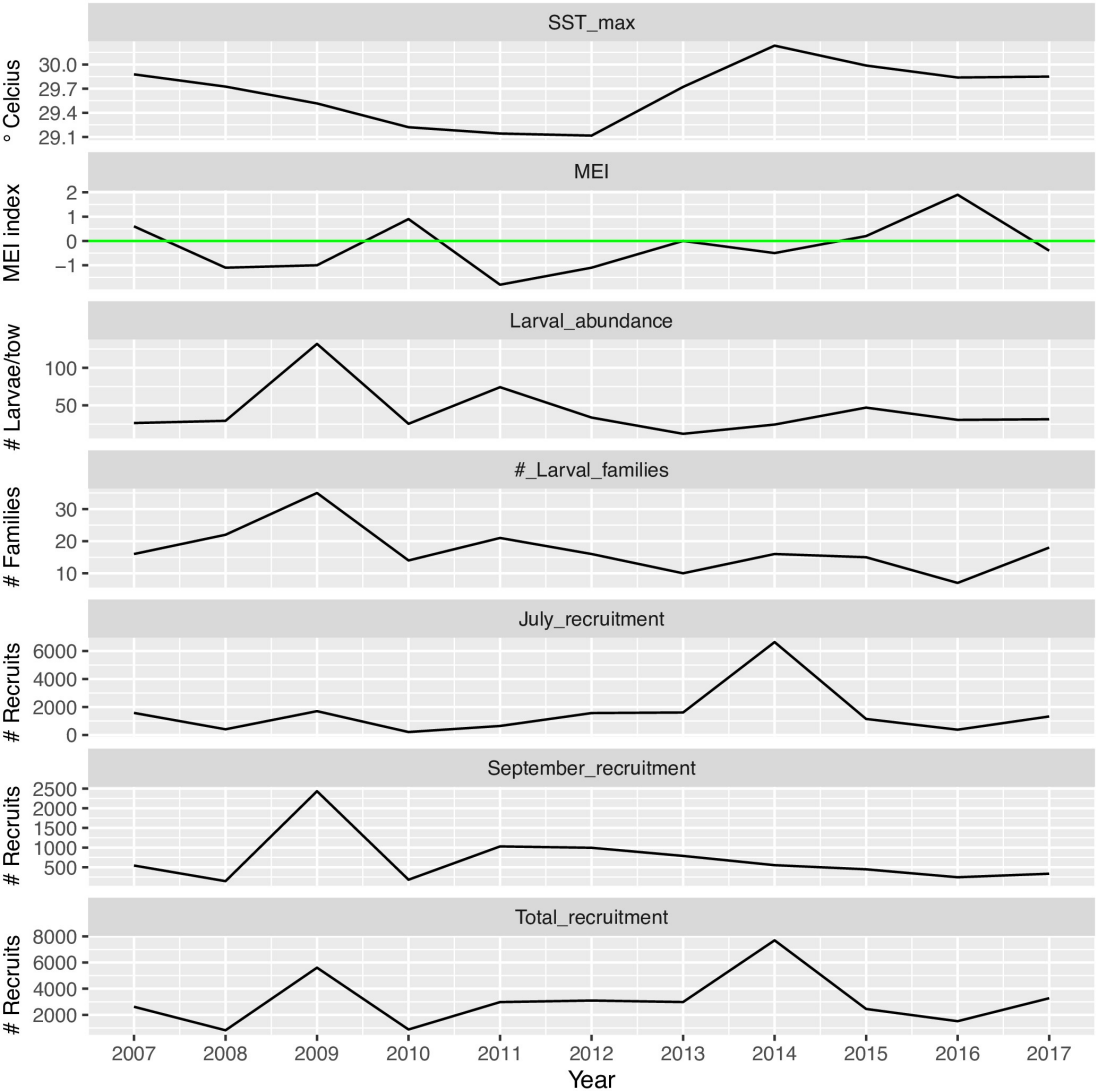
Time series of water column variables, larval supply, and the benthic recruitment of reef fishes on the Main Hawaiian Islands between 2007 and 2017. Larval supply peaked in 2009, and was highly correlated with July recruitment, but not September recruitment. There were two episodic recruitment events that occurred in either the La Niña year of 2008–09 or the Neutral year of 2013–14. Average sea surface temperatures (SST) exceeded 30 d C in the summer of 2014 and remained high through the summer of 2017. See Fig 2 for the correlation matrix among these variables.

Between 2007 and 2017, the multivariate El Niño Index (MEI) measured in December and January fluctuated between strongly positive during the El Niño events of 2006–07, 2009–10, and 2015–16; strongly negative during the La Niña years of 2007–08, 2008–09, 2010–11; and weakly positive/negative during the two Neutral years of 2013–14 and 2014–15 (Fig 1). Of the three El Niño events that occurred during the sampling years, only the 2015–16 El Niño was a classic “EP” type event that originated in the Eastern Pacific; while the remaining two El Niños where “CP” events, originating in the Central Pacific [33].

**Fig 2:**
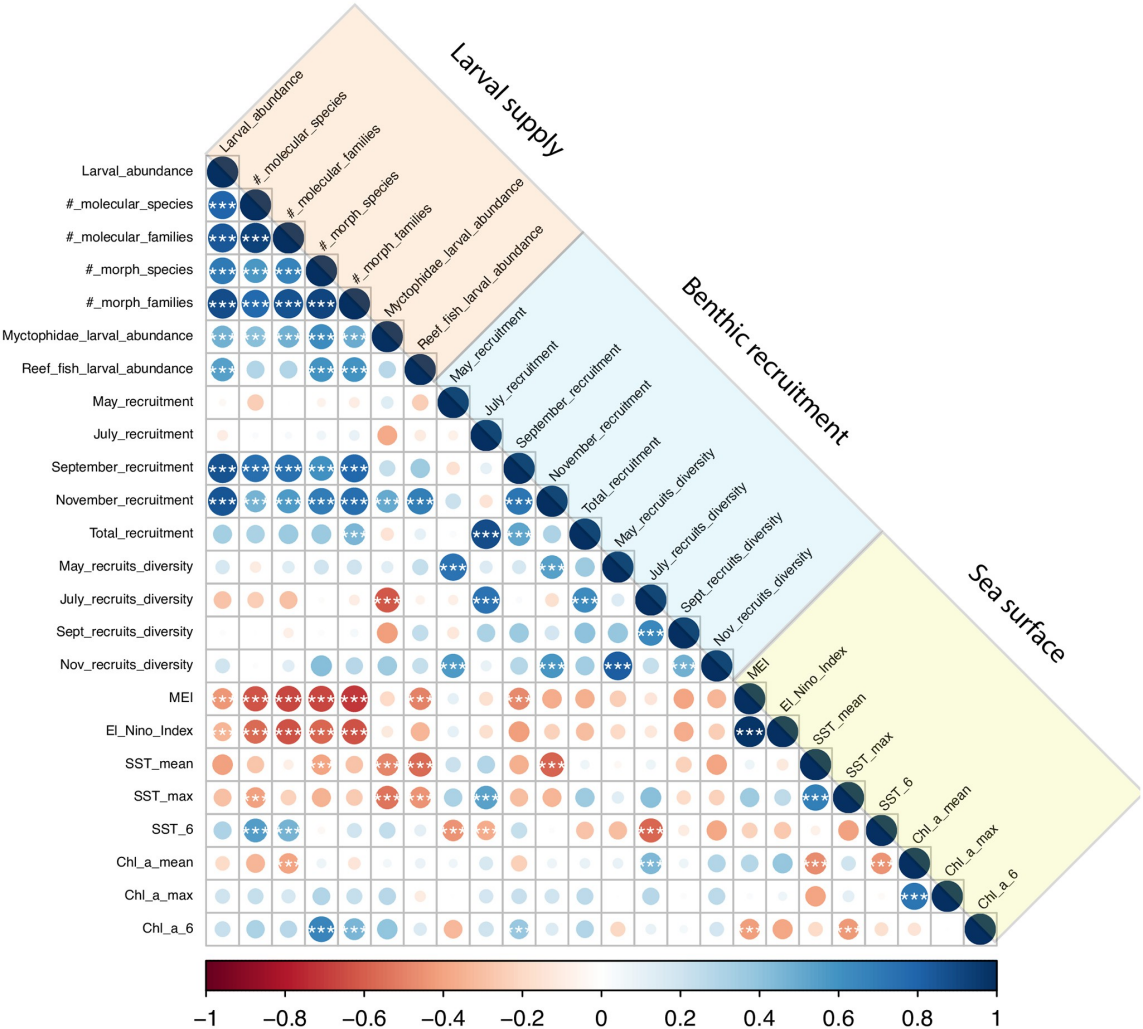
A heat map representation of the correlation matrix of all variables grouped by three categories indicated by colored boxes above the diagonal. Size of the circle, and intensity of color (keyed to color bar), indicates the magnitude of the correlation coefficient. *** indicates coefficients that are statistically significant. For variable definitions, see S2 Table; and for a list of the values of correlation coefficients and their confidence limits, see S4 Table.

Given this dramatic variability in ENSO, we found that the abundance and diversity of larval fishes were negatively correlated with both ENSO indices: the MEI and the El Niño Index (Figs 1 & 2). When the larval data were filtered for species that only belonged to reef-dwelling families, reef fish larval abundance showed a significant negative correlation with MEI (Fig 2). Of reef fish families, the gobys (Gobiidae) tended to dominate the samples (S7 Table). Reef fishes represented 1/3 of all collected samples, with the remainder in epi- and meso-pelagic families but also some deep-sea specialists, such as the dragonfishes (Stomiidae). In contrast to the reef fishes, larval abundance of the mesopelagic fishes in the family Myctophidae did not show significant correlation with MEI, although the sign of the correlation coefficient was also negative. A detailed summary of larval sampling by year and taxonomic group identified by DNA barcoding, can be found in S3 Table.

The strong negative correlation between larval diversity and MEI remained significant after including the variation in sampling effort among years. Partial correlation models that included the number of tows as a covariate, revealed that species diversity, and family diversity were strongly negatively correlated with MEI: all four partial *r* values were significantly greater than 0.0 (Table 1).

**Table 1:** Results from partial correlation models between measures of larval diversity and MEI while controlling for sampling effort. Pearson correlation (*r*), partial correlation coefficients (partial *r*), and the test of significance of partial *r* (P).

Larval diversity variable	*r*	Partial *r*	P
#_molec_species	-0.628	-0.747	0.013
#_molec_families	-0.663	-0.809	0.005
#_morph_species	-0.667	-0.682	0.030
#_morph_families	-0.707	-0.765	0.010

Variables related to larval abundance and diversity were positively correlated with fall recruitment (September and November) on the Big Island of Hawaii, but these same larval variables showed surprisingly weak correlations with surveys of spring and summer recruitment (May and July, Figs 1 & 2). Larval supply and Fall recruitment have declined since 2011 and are associated with increasing maximal SST values measured during this same time frame (Fig 1). There was no significant correlation between MEI and maximal SST around the Main Hawaiian Islands (Figs 1 & 2).

The years of 2009 and 2014 were exceptional with respect to benthic recruitment, with over 5000 benthic recruits counted over the season in each of these years. Both years were characterized by a negative MEI index during the preceding winter months (Fig 1), but the 2014 value was weakly negative in December 2013 and January 2014, resulting in a “Neutral” ENSO phase. During these exceptional recruitment years, two species combined (the yellow tang *Zebrasoma flavescens* and goldeye surgeonfish *Ctenochaetus strigosus*) represented 72% of the total recruitment in 2009 and 62% of the total recruitment in 2014. Benthic recruitment during all sampling months (except June) was positively correlated with species diversity in recruits (Fig 2), indicating that the assemblage of fish species is co-varying with overall recruit abundance, and that the two common species are not solely driving the recruit abundance patterns.

Sea surface temperature (SST mean and maximum) was positively correlated with early season recruitment (May and July) but was negatively correlated with late-season recruitment (September and November) (Fig 2). While this general pattern is striking in the correlation matrix, we note that only two of the eight possible correlations were significant by bootstrapping (July recruitment and SST maximum: r = 0.540, and November recruitment and SST mean, r = -0.586). SST (mean and maximum) was significantly negatively correlated with larval abundance of reef fish and mesopelagic fishes. Few of the other metrics of larval abundance were significantly correlated with SST variables, but the signs were generally negative.

The mean Chl *a* concentration during the six months preceding larval sampling (Chl_a_6) was positively correlated with some larval supply metrics and features of the water column. For example, larval species and family diversity measured by morphology were both positively correlated to Chl_a_6. The MEI and the SST maximum were both negatively correlated with Chl_a_6 (Fig 2). Further, average SST was negatively correlated with average Chl *a*.

### Seasonal temperature and chlorophyl trends

**Fig 3:**
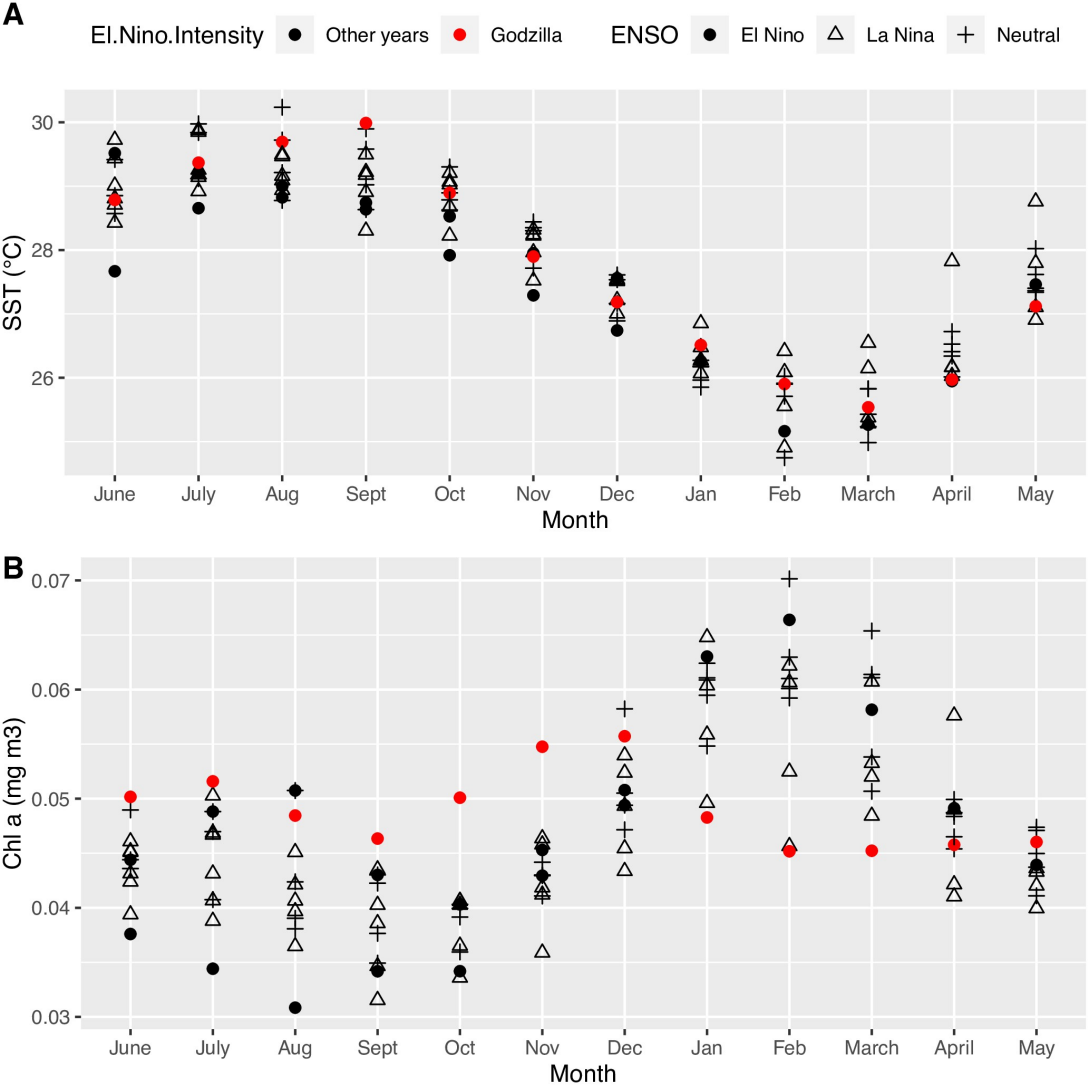
Seasonal patterns of SST (A) and average Chl-*a* biomass (B) around the Main Hawaiian Islands from satellite data between 2006 to 2017. Symbol shapes are keyed to ENSO phases, and red filled circles are for the “Godzilla” El Niño year– 2015–2016 which was a classic “EP” type El Niño [34], while black filled circles are for the lower intensity “CP” type El Niño years (2006–2007; 2009–2010). SST patterns were generally not exceptional for all El Niño events, but during 2015–16 event the SST maximum was high, and the seasonality in chlorophyll biomass was reduced.

A comparison of seasonal trends in mean SST and Chl-*a* around the MHI during sampling years revealed that the 2015–2016 El Niño was exceptional in the Main Hawaiian Islands with respect to maximum water temperature in September (Fig 3A) and in the marked shift in peak chlorophyll biomass to November–December from the more typical peak in February (Fig 3B, contrast the red circles with black symbols). In contrast, there was no clear ENSO signal in the seasonal maximal temperature or Chl-*a* dynamics (Fig 3A & 3B).

### Larval sampling and barcoding statistics

During the ten years of data collection, we sampled a total of 3,645 fish larvae, and annual sample sizes ranged between 153 and 791 larvae (S5 Table). The proportion of the total sample of larvae sequenced each year ranged from 0.64 to 0.12, with a median value of 0.33. Of the sequenced larvae, the median proportion of larvae identified to species with the Barcode of Life Data System (v4) was 0.87, and the median proportion of larvae identified to family by this database was 0.95 (S5 Table). For visual identifications, the median proportion of larvae identified to species was 0.31 and the median proportion identified to family was 0.72 (S5 Table). In general, the visual and barcoding methods produced similar patterns of diversity across years. We sampled a total of 62 families representing a diversity in ecology and depth-related habitats, including pelagic and demersal families that occur near coasts and on coral reefs, but also offshore families, some that are highly migratory, and range in specialization from shallow (epipelagic) to very deep (hadal-pelagic) depths. A summary of family diversity by morphological identification sampled each year is given in S3 Table. All barcoding sequences are available in the Barcode of Life Data System v4, with the project code FLHI. Sequences were also submitted to GenBank, under the accession #s PP965796-PP966901.

## Discussion

To our knowledge, this study is the first to quantify the dynamics of the supply of larval fishes in Hawaii across multiple El Niño and La Niña events. We have brought together three unique time series to show a coupling between ENSO and late winter larval supply. We found the highest abundance and diversity of larval fishes when the MEI index was negative (La Niña conditions) while lowest abundance and diversity occurred when the MEI index was positive (El Niño conditions). The linkage between larval supply in the water column near Oahu and benthic recruitment on coral reefs on the West Coast of the Big Island varied with the month of recruitment. Specifically, we found strong linkage between larval supply and benthic recruitment during the Fall months (September and November), but unexpectedly weak linkages between larval supply and recruitment during the Spring and Summer months (May and July). Given that average planktonic larval durations in reef fishes range between one and three months [10, 35], we would expect that larval supply in late winter to be more strongly correlated with spring or summer recruitment. This suggests that the planktonic larval pool is likely to be spatially heterogenous within and between islands. Nonetheless, the largest pulses of benthic recruitment occurred during the summers that followed winters of the strong La Niña event of 2008–09 and the Neutral event of 2013–14, Our larval samples clearly indicate that these non-El Niño phases of ENSO are generally favorable for successful larval development and transport back to adult habitats. Fox et al. [25] analyzed earlier years of the WHAP data set (1999–2010) and also found a pattern of the largest recruitment pulses to occur after La Niña or Neutral conditions. This study reported three unusually large recruitment years where greater than 6000 recruits were counted: 2002 (Dec.–Jan. MEI = 0.07), 2005 (Dec.- Jan. MEI = 0.08), and 2009 (Dec.- Jan. MEI = -1.01). Using a time-lag analysis (autocorrelation), Fox et al. also found a negative relationship between winter water temperature and benthic recruitment measured six months later, a result that is consistent with our finding of a truncated summer recruitment season during the high SSTs recorded during the successive 2014–15 and 2015–16 El Niño events. It is also important to point out that the WHAP data set on benthic recruitment is dominated by surgeonfishes (Acanthuridae), while the most abundant reef fish in our larval samples from Oahu were gobies (Gobiidae). Exceptional swimming speeds by the larvae of Acanthurid species (see details below) probably decrease their capture in plankton nets, but the gobies and other reef fish taxa provide a reasonable proxy for onshore transport of the nearshore species in Hawaii. Collectively, these results suggests that physical and biological conditions for the development and transport of larvae to adult habitats in the Main Hawaiian Islands are favorable during the Neutral or La Niña phases of ENSO but unfavorable during El Niño phases.

**Fig 4:**
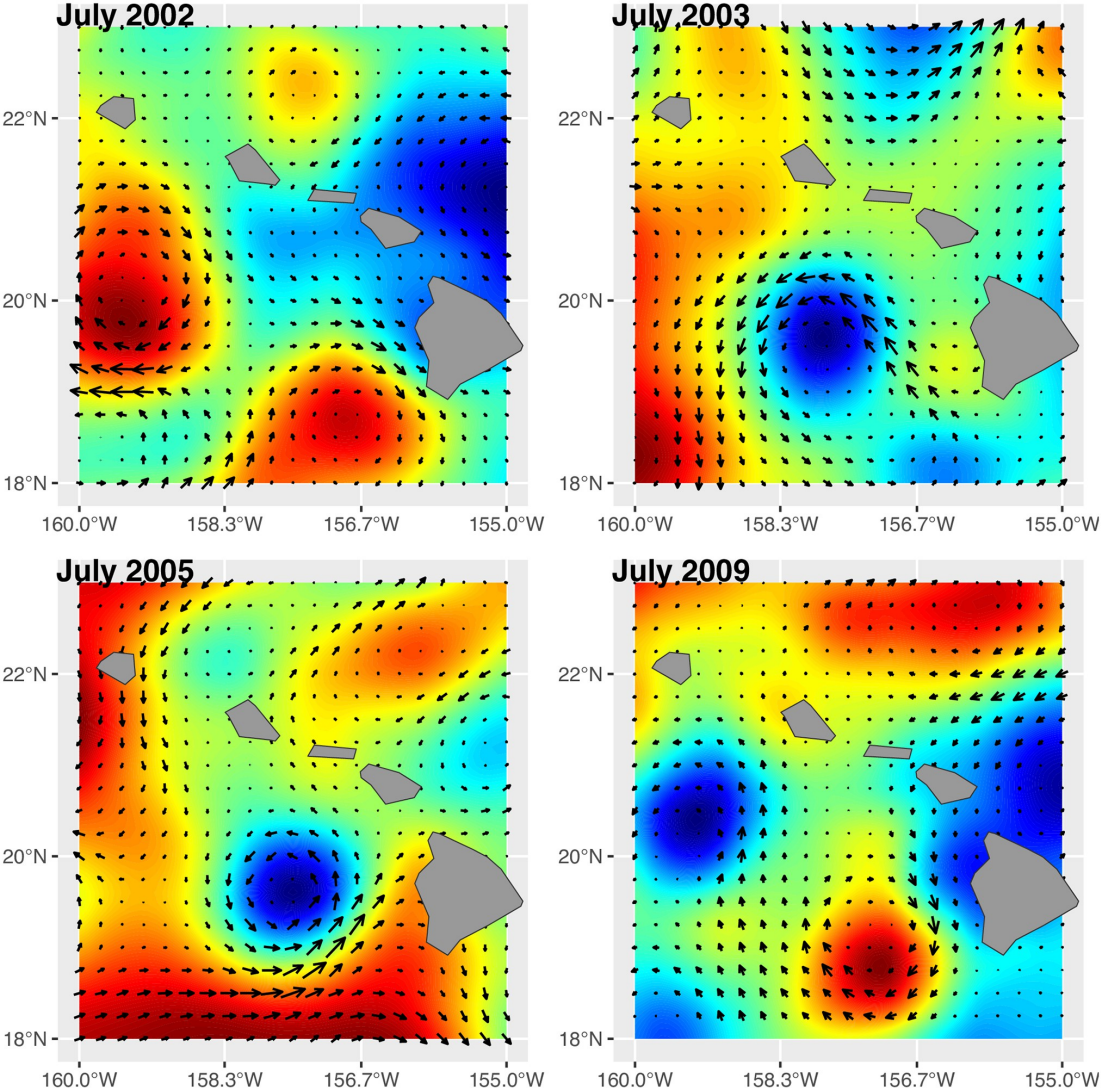
Eddie formation from AVHRR data near the Main Hawaiian Islands during July for years of exceptional coral reef fish recruitment (2002, 2005, and 2009) and a year with lower recruitment (2003). Note that all the years with exceptional coral reef fish recruitment on the West Coast of the Big Island were characterized by the juxtaposition of anticylonic (blue) and cyclonic (red) eddies with a net effect of accelerating larval transport onshore. In contrast, the average recruitment year of 2003 was characterized by eddies with the net effect of transporting larvae offshore. High recruitment years (‘02, ‘05, ‘09) coincided with neutral or La Nina conditions starting the preceding late winter, while the lower recruitment year (‘03) had strong El Nino conditions during the preceding late winter.

The physiology of larval fishes, and the seasonality of spawning dynamics in the Hawaiian Islands [26], suggests that the linkage between ENSO and larval supply in the late winter and spring months is not related to increased temperature stress during El Niño phases. Larvae that were developing in the water column the coasts of the Hawaiian Islands during the period of January through April experienced average temperatures well below 28°C (Fig 3a), and below the heat stress thresholds of 30–32°C reported for the larvae of other reef fishes [16, 36]. In fact, temperatures below these upper stress thresholds are typically positively related to larval physiology and performance in other coral reef fishes [37], and there was a positive, albeit not significant, relationship between average SST measured 6 months prior to larval collection (SST_6) and our larval abundance and diversity measures. On the other hand, later in the spawning season, in August and September, the potential limitations of high temperature become clear in our data. The SST maxima for the years between 2014 and 2017 exceeded 30°C in the late summer (Figs 1 and 3a), and these years all had very low levels of recruitment during the late summer and fall sampling months. These temperature patterns are decoupled from the ENSO cycle, as high SSTs between 2014 and 2017 occurred in both Neutral and El Niño phases. Perhaps the most intriguing result of our collective analysis is the fact that higher SST maxima appear to be constraining the temporal window for successful larval development and recruitment. One of the largest pulses of recruitment on the Big Island occurred during 2014 when the MEI index was near 0 (Neutral phase) but the SST maximum exceeded 30°C. During this year, nearly all the recruitment occurred well before this maximum was reached, during the months of May and July (Fig 1, July vs. September recruitment). The apparent positive effect of average and maximum SST on benthic recruitment during the early season, which turns negative during the late season, is illustrated by the sign of the correlation coefficients between these two temperature variables and recruitment measured among the four months. In the early season (May & July), the correlation is positive, but in the late season (September & November) the correlation turns negative (see correlation matrix, Fig 2). Regardless of the phase of the ENSO cycle, sea surface temperature (SST) maxima during the late summer and fall are negatively related to benthic recruitment on the Big Island. The pattern of high larval abundance and high recruitment that occurs during some La Niña or Neutral phases, but not in El Niño phases, is most likely the product of a complex interaction between water column conditions that favor the feeding and development of larval fishes and the successful transport of these larvae back to nearshore habitats. Our measurements of larval supply in the early part of the spawning season suggest that conditions for larval growth and survivorship are positive during the La Niña phase of ENSO but that benthic recruitment is more stochastic with respect to La Niña or Neutral phases. One explanation for stochastic recruitment during these ENSO phases is ultimately related to the dynamics of mesoscale eddies that form to the west-southwest of the Main Hawaiian Islands. These rotating cyclonic and anticyclonic eddies, 50–150 km in diameter, are generated by wind stress patterns associated with trade-winds that are blowing over and between the Main Hawaiian Islands [38, 39]. Eckman circulation within cyclonic eddies drives upwelling of cooler, nutrient-rich water, dramatically increasing productivity compared to surrounding oligotrophic waters [40–42] (Fig 4). Innovative experimental studies in the Caribbean Sea have shown that larval growth and survivorship can be several times greater inside of eddies than outside these features [43, 44].

Cyclonic eddies have long been hypothesized to entrain larval fishes and potentially advect them towards the shores of the Main Hawaiian Islands [45], but the complex relationship between mesoscale eddy dynamics and their impacts on larval recruitment in Hawaii has remained challenging to unravel. A previous study in Hawaii did not find any strong relationships between the frequency of cyclonic eddy formation within years and the number of coral reef fish recruits on the Big Island using the West Hawaii Aquarium Project data from 1999–2010 [25]. A recent modeling analysis has shown that retention and transport of larvae back to shore is more likely in cyclonic vs. anticyclonic mesoscale eddies [46], and a climatic analysis of CP and EP type El Niño’s since 1957 reveals that trade wind strength decreases particularly in Eastern Pacific events (as in 2015/16) and to a lesser degree in Central Pacific events [34]. Thus, in general, stronger trade-winds during La Niña and Neutral events should be favorable for eddy formation. The pattern of juxtaposed cold and warm-core eddies during La Niña/Neutral years, that coincided with high recruitment years (Fig 4), implicates a strong role of physical oceanography in successful recruitment. Larval fishes would benefit from the higher productivity of cold core eddies that is coupled with transport back to reef habitats in currents that are generated by pairs of cold- and warm-core eddies spinning in opposite directions. In this scenario, the structure of eddies in relation to the Hawaiian Islands is as important as the frequency of eddy development.

We have emphasized a paradigm of decoupling between reproduction and larval supply that is driven by the different habitats used by adult and larval fishes, the length of time to complete development in the plankton, and the relatively slow swimming speeds of developing larvae [47]. On the other hand, the planktonic larvae of some reef fishes have several remarkable adaptations that can increase their retention near natal reefs and the probability of returning to suitable nearshore habitat and even the natal reef. For example, the larvae of a diversity of reef fish species, including the surgeonfishes (Acanthuridae), can use sound to navigate towards suitable reef habitats [48, 49]. Further, the surgeonfishes have the highest swimming speeds recorded of any tropical fish family, with average swimming speeds that exceed 40 cm s^-1^ [50]. A recent parentage analysis conducted on the reefs of Oahu, provides support for the idea that a significant fraction of surgeonfish larvae can navigate back to their home reef [51]. In this study, over 600 *Acanthurus triostegus sandvicensis* juveniles were genotyped, and 11.2% of this sample was assigned to the same reef as their parents. If larvae of the two dominant acanthurid species on the Big Island: *Zebrasoma flavescens* and *Ctenochaetus strigosus*; also employ behavior to increase retention near natal reefs, then the supply of new recruits to benthic populations will be connected to benthic processes that mediate adult reproductive output in natal habitats as well as those processes acting on larvae in surface waters [52]. Since temperatures that exceed average seasonal maxima have been shown to negatively impact reproduction in tropical fishes [11], the negative relationship we observed between SST maxima and benthic recruitment during the summer months could be a signal that temperature is impacting benthic adults as well planktonic larval phases.

Determining the impacts of ENSO climate oscillations on larval supply and larval recruitment requires appropriate longitudinal sampling on the scale of decades, a temporal sampling scale which is rare in studies of larval fishes (but see [53]). In the tropics and subtropics only a handful of longitudinal studies have examined the impact of ENSO on larval fishes, and these have been focused on relatively short temporal scales, for example, a single ENSO event [18, 54, 55]. None to our knowledge that have sampled both pelagic larvae and benthic recruits across multiple ENSO events. The intriguing correlations revealed by our analyses of time series data related to the dynamics of the supply and recruitment of larval fishes reveal that the Hawaiian Islands are a particularly rich system to further understand how climatic oscillations such as ENSO are having fundamental impacts on tropical marine systems. A limitation of our planktonic data set is that the sampling dates and locations were tied to the timing of an undergraduate laboratory exercise at the University of Hawaii, Manoa. This constrained our temporal sampling to the beginning of the reproductive season and limited cruises to a single location near Honolulu. As in other regions of the world’s oceans, there is clearly more scope for temporally and spatially explicit data sets of larval fishes that are capable of detecting shifts in seasonal phenology and changes in abundance and diversity on decadal scales. We have shown the utility of larval barcoding for providing species-specific identification that could also potentially be scaled up to larger sampling designs. These larval and benthic time series, when coupled with an expanding understanding of the oceanography around the Hawaiian Islands [56, 57], hold much promise of establishing a more detailed view of the crucial links between larval production, larval supply, and benthic recruitment; but also how an increasing extreme environment is altering these links in diverse and productive tropical marine ecosystems.

## Supporting information

S1 Table The sampling details of the plankton tows. (DOCX)

S2 Table A description of all variables used in the correlation matrix. (DOCX)

S3 Table The number of larvae identified by DNA barcoding in 62 fish taxa sampled by year, from 2007–2017. Asterisks (*) indicate families with species that occur primarily nearshore and are associated with coral reefs. The total number of larvae sampled in these taxa were used for estimates of reef fish larval abundance/year.
(DOCX)

S4 Table Bootstrapped 95% confidence limits of correlation coefficients. (XLSX)

S5 Table Estimates of species and family diversity by year based on DNA barcoding and visual identification. (DOCX)

S6 Table Barcode of Life Database (BOLD) identifications for all sequenced larvae. Sample.ID–BOLD catalogue number, %Match–The % of matching sequence between larval sequence and BOLD matching sequence, Bp overlap–overlap in nucleotide base pair between larval sequence and BOLD matching sequence. Other column headers are self-explanatory.
(XLSX)

S7 Table Results from the visual identifications of the entire data set. Rows 4–52 are species level identifications for each year and each depth. Rows 57–132 are family identifications (and in some cases genus-level) for each year and each depth. The total number of larvae that could not be identified at the family level are listed in Row 135. Taxa in red font were included in the reef fishes functional group for the correlation analysis (Fig 2).
(XLSX)
