# Supplementary material for: Coupling and de-coupling of the El Niño Southern Oscillation to the supply of larval fishes to benthic populations in the Hawaiian Islands
Source: PLoS One. 2024 Oct 24;19(10):e0312593. doi: 10.1371/journal.pone.0312593 (PMC11500875; doi:10.1371/journal.pone.0312593)
Supplement: S1 Table — (DOCX) [file pone.0312593.s001.docx]

S1 Table. The sampling details of the plankton tows.

| Year | Day | Depth | Duration (min) | Volume (m^3) | Vessel |
| --- | --- | --- | --- | --- | --- |
| 2007 | 16-Jan | 1 | N/A | N/A | Klaus Wyrtki |
| 2007 | 16-Jan | 25 | N/A | N/A | Klaus Wyrtki |
| 2007 | 16-Jan | 50 | N/A | N/A | Klaus Wyrtki |
| 2007 | 17-Jan | 1 | N/A | N/A | Klaus Wyrtki |
| 2007 | 17-Jan | 25 | N/A | N/A | Klaus Wyrtki |
| 2007 | 17-Jan | 50 | N/A | N/A | Klaus Wyrtki |
| 2008 | 22-Jan | 1 | N/A | N/A | Klaus Wyrtki |
| 2008 | 22-Jan | 25 | N/A | N/A | Klaus Wyrtki |
| 2008 | 22-Jan | 50 | N/A | N/A | Klaus Wyrtki |
| 2008 | 23-Jan | 1 | N/A | N/A | Klaus Wyrtki |
| 2008 | 23-Jan | 25 | N/A | N/A | Klaus Wyrtki |
| 2008 | 23-Jan | 50 | N/A | N/A | Klaus Wyrtki |
| 2008 | 24-Jan | 1 | N/A | N/A | Klaus Wyrtki |
| 2008 | 24-Jan | 25 | N/A | N/A | Klaus Wyrtki |
| 2008 | 24-Jan | 50 | N/A | N/A | Klaus Wyrtki |
| 2009 | 20-Jan | 1 | 20 | 829.19 | Klaus Wyrtki |
| 2009 | 20-Jan | 25 | 17 | 647.43 | Klaus Wyrtki |
| 2009 | 20-Jan | 50 | 26 | N/A | Klaus Wyrtki |
| 2009 | 21-Jan | 1 | N/A | 832.95 | Klaus Wyrtki |
| 2009 | 21-Jan | 25 | N/A | N/A | Klaus Wyrtki |
| 2009 | 21-Jan | 50 | N/A | 662.96 | Klaus Wyrtki |
| 2010 | 19-Jan | 1 | N/A | N/A | Klaus Wyrtki |
| 2010 | 19-Jan | 25 | N/A | N/A | Klaus Wyrtki |
| 2010 | 19-Jan | 50 | N/A | N/A | Klaus Wyrtki |
| 2010 | 20-Jan | 1 | N/A | N/A | Klaus Wyrtki |
| 2010 | 20-Jan | 25 | N/A | N/A | Klaus Wyrtki |
| 2010 | 20-Jan | 50 | N/A | N/A | Klaus Wyrtki |
| 2011 | 18-Jan | 1 | 18 | N/A | Klaus Wyrtki |
| 2011 | 18-Jan | 25 | 22 | N/A | Klaus Wyrtki |
| 2011 | 18-Jan | 50 | 24 | N/A | Klaus Wyrtki |
| 2011 | 19-Jan | 1 | N/A | N/A | Klaus Wyrtki |
| 2011 | 19-Jan | 25 | N/A | N/A | Klaus Wyrtki |
| 2011 | 19-Jan | 50 | N/A | N/A | Klaus Wyrtki |
| 2011 | 20-Jan | 1 | N/A | N/A | Klaus Wyrtki |
| 2011 | 20-Jan | 25 | N/A | N/A | Klaus Wyrtki |
| 2011 | 20-Jan | 50 | N/A | N/A | Klaus Wyrtki |
| 2012 | 27-Feb | 1 | 18 | 497.70 | Huki Pono |
| 2012 | 27-Feb | 25 | 18 | 449.64 | Huki Pono |
| 2012 | 27-Feb | 50 | 24 | 471.30 | Huki Pono |
| 2012 | 28-Feb | 1 | 19 | 561.63 | Huki Pono |
| 2012 | 28-Feb | 25 | 18 | 611.42 | Huki Pono |
| 2012 | 28-Feb | 50 | 31 | 813.91 | Huki Pono |
| 2012 | 1-Mar | 1 | 10 | 418.53 | Huki Pono |
| 2012 | 1-Mar | 25 | 17 | 460.58 | Huki Pono |
| 2012 | 1-Mar | 50 | 10 | 444.09 | Huki Pono |
| 2012 | 29-Feb | 1 | 17 | 431.20 | Huki Pono |
| 2012 | 29-Feb | 25 | 22 | 389.79 | Huki Pono |
| 2012 | 29-Feb | 50 | 20 | 389.76 | Huki Pono |
| 2013 | 5-Feb | 1 | 10 | 260.32 | Hookele |
| 2013 | 5-Feb | 1 | 13 | 540.29 | Hookele |
| 2013 | 5-Feb | 25 | 10 | 242.95 | Hookele |
| 2013 | 5-Feb | 25 | 14 | 376.21 | Hookele |
| 2013 | 5-Feb | 50 | 14 | 288.77 | Hookele |
| 2013 | 5-Feb | 50 | 16 | 349.20 | Hookele |
| 2013 | 6-Feb | 1 | 10 | 327.65 | Hookele |
| 2013 | 6-Feb | 1 | 11 | 310.01 | Hookele |
| 2013 | 6-Feb | 25 | 11 | 161.23 | Hookele |
| 2013 | 6-Feb | 25 | 13 | 242.30 | Hookele |
| 2013 | 6-Feb | 50 | 14 | 162.20 | Hookele |
| 2013 | 6-Feb | 50 | 13 | 244.43 | Hookele |
| 2013 | 7-Feb | 1 | 10 | 337.93 | Hookele |
| 2013 | 7-Feb | 25 | 10 | 166.63 | Hookele |
| 2013 | 7-Feb | 50 | 10 | 180.44 | Hookele |
| 2014 | 11-Feb | 1 | 15 | 584.34 | Hookele |
| 2014 | 11-Feb | 1 | 20 | 719.12 | Hookele |
| 2014 | 11-Feb | 25 | 12 | 556.21 | Hookele |
| 2014 | 11-Feb | 25 | 10 | 433.05 | Hookele |
| 2014 | 11-Feb | 50 | 16 | 616.00 | Hookele |
| 2014 | 11-Feb | 50 | 13 | 506.71 | Hookele |
| 2014 | 12-Feb | 1 | 17 | 758.11 | Hookele |
| 2014 | 12-Feb | 1 | 16 | 567.73 | Hookele |
| 2014 | 12-Feb | 25 | 19 | 690.19 | Hookele |
| 2014 | 12-Feb | 25 | 19 | 704.31 | Hookele |
| 2014 | 12-Feb | 50 | 24 | 844.18 | Hookele |
| 2014 | 12-Feb | 50 | 22 | 761.23 | Hookele |
| 2014 | 13-Feb | 1 | 15 | 590.76 | Hookele |
| 2014 | 13-Feb | 25 | 15 | 612.62 | Hookele |
| 2014 | 13-Feb | 50 | 15 | 590.97 | Hookele |
| 2015 | 27-Jan | 1 | 15 | 166.36 | Searcher |
| 2015 | 27-Jan | 25 | 23 | N/A | Searcher |
| 2015 | 27-Jan | 50 | 44 | 321.34 | Searcher |
| 2015 | 28-Jan | 1 | 15 | N/A | Searcher |
| 2015 | 28-Jan | 25 | 25 | 219.57 | Searcher |
| 2015 | 28-Jan | 50 | 33 | 195.84 | Searcher |
| 2016 | 26-Jan | 1 | 12 | 165.56 | Searcher |
| 2016 | 26-Jan | 25 | 24 | 282.59 | Searcher |
| 2016 | 26-Jan | 50 | 10 | 126.30 | Searcher |
| 2016 | 27-Jan | 1 | 21 | 214.77 | Searcher |
| 2016 | 27-Jan | 25 | 20 | 380.52 | Searcher |
| 2016 | 27-Jan | 50 | 38 | 334.83 | Searcher |
| 2017 | 25-Jan | 1 | 31 | N/A | Searcher |
| 2017 | 25-Jan | 25 | 19 | 193.42 | Searcher |
| 2017 | 25-Jan | 50 | 37 | 797.87 | Searcher |
| 2017 | 26-Jan | 1 | 28 | 407.85 | Searcher |
| 2017 | 26-Jan | 25 | 27 | 363.30 | Searcher |
| 2017 | 26-Jan | 50 | 30 | 487.27 | Searcher |
