# Supplementary material for: Coupling and de-coupling of the El Niño Southern Oscillation to the supply of larval fishes to benthic populations in the Hawaiian Islands
Source: PLoS One. 2024 Oct 24;19(10):e0312593. doi: 10.1371/journal.pone.0312593 (PMC11500875; doi:10.1371/journal.pone.0312593)
Supplement: S2 Table — (DOCX) [file pone.0312593.s002.docx]

S2 Table. A description of all variables used in the correlation matrix.

| Variable | Description |
| --- | --- |
| Larval abundance | The total number of fish larvae sampled per year / # of tows made that year |
| #_molec_species | The number of species in the sample. The species of each sample identified by DNA barcoding. |
| #_molec_families | The number of families in the sample. The family of each sample identified by DNA barcoding. |
| #_morph_species | The number of species in the sample. The species of each sample identified by morphology. |
| #_morph_families | The number of families in the sample. The family of each sample identified by morphology. |
| Myctophidae_larval_abundance | The total number of larval fishes from the family Myctophidae (lanternfishes) identified by morphology / # of tows made that year |
| Reef_fish_larval_abundance | The total number of larval fishes from families associated with near shore habitats or coral reefs identified by morphology / # of tows made that year. See S3 Table for the specific families included in this group |
| May_recruitment | The total number of recruits counted in May, WHAP surveys |
| July_ recruitment | The total number of recruits counted in July, WHAP surveys |
| September_recruitment | The total number of recruits counted in September, WHAP surveys |
| October_recruitment | The total number of recruits counted in October, WHAP surveys |
| Total_recruitment | The total number of recruits counted for the year, WHAP surveys |
| May_recruits_diversity | The total number of species observed in May, WHAP surveys. |
| July_recruits_diversity | The total number of species observed in July, WHAP surveys. |
| September_recruits_diversity | The total number of species observed in September, WHAP surveys. |
| October_recruits_diversity | The total number of species observed in October, WHAP surveys. |
| MEI | The multivariate El Nino index in January |
| El_Nino_Index | The El Nino 3.4 anomaly in January |
| SST_mean | The average annual sea surface temperature across the Main Hawaiian Islands grid. |
| SST_max | The maximum annual sea surface temperature from the Main Hawaiian Islands grid. |
| SST_6 | Average sea surface temperature calculated from the six months preceding larval sampling across the Main Hawaiian Islands grid. |
| Chl_a_mean | The average annual biomass of chl *a* (mg m^3^) across the Main Hawaiian Islands grid. |
| Chl_a_min | The minimum annual biomass of chl *a* (mg m^3^) across the Main Hawaiian Islands grid. |
| Chl_a_ max | The maximum annual biomass of chl *a* (mg m^3^) across the Main Hawaiian Islands grid. |
| Chl_a_6 | The average biomass of chl *a* (mg m^3^) calculated from the six months preceding larval sampling across the Main Hawaiian Islands grid. |
| Chl_12 | The average biomass of chl *a* (mg m^3^) calculated from the 12 months preceding larval sampling across the Main Hawaiian Islands grid. |
