# Supplementary material for: Coupling and de-coupling of the El Niño Southern Oscillation to the supply of larval fishes to benthic populations in the Hawaiian Islands
Source: PLoS One. 2024 Oct 24;19(10):e0312593. doi: 10.1371/journal.pone.0312593 (PMC11500875; doi:10.1371/journal.pone.0312593)
Supplement: S3 Table — Asterisks (*) indicate families with species that occur primarily nearshore and are associated with coral reefs. The total number of larvae sampled in these taxa were used for estimates of reef fish larval abundance/year. (DOCX) [file pone.0312593.s003.docx]

S3 Table. The number of larvae identified by DNA barcoding in 62 fish taxa sampled by year, from 2007 – 2017. Asterisks (*) indicate families with species that occur primarily nearshore and are associated with coral reefs. The total number of larvae sampled in these taxa were used for estimates of reef fish larval abundance/year.

| Row Labels | 2007 | 2008 | 2009 | 2010 | 2011 | 2012 | 2013 | 2014 | 2015 | 2016 | 2017 | All years |
| --- | --- | --- | --- | --- | --- | --- | --- | --- | --- | --- | --- | --- |
| Acanthuridae* | 0 | 0 | 2 | 0 | 0 | 0 | 0 | 0 | 0 | 1 | 0 | 3 |
| Anguiliformes* | 0 | 1 | 1 | 0 | 5 | 0 | 0 | 2 | 1 | 2 | 0 | 12 |
| Apogonidae* | 0 | 5 | 1 | 3 | 15 | 9 | 0 | 14 | 1 | 4 | 0 | 52 |
| Atherinidae | 0 | 1 | 1 | 0 | 0 | 0 | 0 | 0 | 0 | 0 | 0 | 2 |
| Aulostomatidae* | 0 | 0 | 0 | 0 | 0 | 0 | 0 | 0 | 1 | 0 | 0 | 1 |
| Blenniidae* | 0 | 10 | 14 | 2 | 3 | 0 | 2 | 12 | 4 | 0 | 4 | 51 |
| Blenniidae-  Salariinii* | 0 | 2 | 14 | 11 | 0 | 8 | 7 | 7 | 8 | 2 | 6 | 65 |
| Bothidae | 0 | 1 | 5 | 1 | 0 | 0 | 0 | 0 | 0 | 0 | 0 | 7 |
| Bramidae | 0 | 0 | 2 | 0 | 1 | 0 | 0 | 0 | 1 | 0 | 1 | 5 |
| Bregmacerotidae | 1 | 0 | 0 | 0 | 1 | 0 | 0 | 2 | 0 | 1 | 0 | 5 |
| Callionymidae | 0 | 1 | 4 | 4 | 22 | 11 | 2 | 2 | 1 | 1 | 0 | 48 |
| Carangidae* | 3 | 38 | 6 | 0 | 0 | 0 | 0 | 14 | 4 | 0 | 1 | 66 |
| Ceratiidae | 0 | 0 | 1 | 0 | 0 | 0 | 0 | 0 | 0 | 0 | 0 | 1 |
| Ceratioidei | 0 | 0 | 0 | 0 | 0 | 0 | 0 | 2 | 0 | 0 | 0 | 2 |
| Chiasmodontidae | 1 | 0 | 1 | 0 | 0 | 0 | 1 | 0 | 0 | 0 | 1 | 4 |
| Cirrhitidae | 1 | 0 | 0 | 0 | 0 | 0 | 0 | 0 | 0 | 0 | 0 | 1 |
| Clupeidae | 0 | 1 | 0 | 0 | 0 | 0 | 0 | 0 | 0 | 0 | 0 | 1 |
| Congridae* | 0 | 0 | 0 | 0 | 1 | 0 | 0 | 0 | 0 | 0 | 0 | 1 |
| Coryphaenidae | 0 | 0 | 1 | 0 | 0 | 0 | 0 | 1 | 0 | 0 | 3 | 5 |
| Creediidae | 0 | 0 | 2 | 0 | 1 | 1 | 0 | 0 | 1 | 0 | 0 | 5 |
| Dussumieriidae | 0 | 0 | 0 | 0 | 0 | 0 | 0 | 1 | 0 | 0 | 0 | 1 |
| Engraulidae | 0 | 0 | 0 | 0 | 0 | 1 | 0 | 0 | 0 | 0 | 0 | 1 |
| Evermannellidae | 0 | 0 | 0 | 0 | 1 | 0 | 0 | 0 | 1 | 0 | 0 | 2 |
| Exocoetidae | 0 | 0 | 0 | 4 | 0 | 0 | 1 | 3 | 0 | 0 | 0 | 8 |
| Gempylidae | 0 | 0 | 6 | 0 | 4 | 0 | 0 | 0 | 0 | 0 | 2 | 12 |
| Gobiidae* | 10 | 12 | 29 | 17 | 341 | 89 | 23 | 101 | 36 | 79 | 4 | 741 |
| Gonostomatidae | 0 | 1 | 5 | 6 | 4 | 8 | 20 | 6 | 4 | 1 | 6 | 61 |
| Hemiramphidae | 0 | 0 | 0 | 0 | 0 | 0 | 1 | 0 | 0 | 0 | 0 | 1 |
| Howellidae | 0 | 1 | 1 | 1 | 3 | 0 | 1 | 0 | 1 | 0 | 0 | 8 |
| Kuhliidae | 0 | 0 | 1 | 0 | 0 | 0 | 0 | 0 | 0 | 0 | 0 | 1 |
| Kyphosidae* | 0 | 0 | 0 | 0 | 0 | 0 | 0 | 0 | 0 | 0 | 1 | 1 |
| Labridae* | 4 | 2 | 2 | 1 | 0 | 2 | 3 | 0 | 0 | 0 | 1 | 15 |
| Lutjanidae* | 3 | 0 | 1 | 0 | 1 | 0 | 0 | 0 | 0 | 0 | 0 | 5 |
| Melamphaidae | 0 | 0 | 1 | 1 | 1 | 0 | 1 | 0 | 0 | 0 | 0 | 4 |
| Melanocetidae | 0 | 0 | 0 | 2 | 0 | 0 | 0 | 0 | 6 | 0 | 12 | 20 |
| Microdesmidae | 0 | 0 | 1 | 0 | 1 | 0 | 0 | 0 | 1 | 0 | 0 | 3 |
| Molidae | 22 | 44 | 473 | 4 | 2 | 19 | 11 | 3 | 60 | 0 | 61 | 699 |
| Mullidae | 1 | 6 | 3 | 0 | 0 | 0 | 0 | 0 | 0 | 0 | 3 | 13 |
| Myctophidae | 17 | 46 | 57 | 74 | 88 | 24 | 46 | 38 | 21 | 17 | 46 | 474 |
| Nomeidae | 1 | 0 | 0 | 0 | 1 | 0 | 0 | 0 | 0 | 0 | 0 | 2 |
| Notosudidae | 0 | 0 | 0 | 0 | 0 | 0 | 1 | 0 | 1 | 0 | 0 | 2 |
| Ophidiidae | 1 | 0 | 0 | 1 | 0 | 0 | 0 | 0 | 0 | 0 | 0 | 2 |
| Paralepididae | 0 | 0 | 0 | 0 | 2 | 0 | 0 | 0 | 2 | 0 | 0 | 4 |
| Paralepididae/  Lestidiidae | 0 | 0 | 0 | 0 | 3 | 1 | 0 | 1 | 0 | 1 | 0 | 6 |
| Phosichthyidae | 0 | 1 | 4 | 1 | 6 | 3 | 5 | 1 | 0 | 0 | 0 | 21 |
| Pinguipedidae | 0 | 0 | 0 | 0 | 1 | 0 | 0 | 0 | 0 | 0 | 0 | 1 |
| Pomacanthidae* | 0 | 0 | 0 | 0 | 2 | 0 | 0 | 3 | 0 | 0 | 0 | 5 |
| Pomacentridae* | 2 | 2 | 19 | 0 | 4 | 3 | 0 | 0 | 37 | 1 | 5 | 73 |
| Ptereleotridae | 4 | 0 | 0 | 0 | 1 | 0 | 0 | 0 | 0 | 0 | 0 | 5 |
| Scaridae* | 0 | 0 | 1 | 0 | 0 | 1 | 0 | 0 | 0 | 0 | 0 | 2 |
| Schindleriidae | 0 | 5 | 4 | 0 | 8 | 32 | 2 | 25 | 3 | 22 | 0 | 101 |
| Scombridae | 4 | 9 | 4 | 0 | 0 | 1 | 0 | 0 | 0 | 0 | 0 | 18 |
| Scombrolabracidae | 0 | 0 | 3 | 2 | 3 | 0 | 0 | 2 | 0 | 0 | 1 | 11 |
| Scorpaenidae* | 0 | 0 | 4 | 1 | 0 | 0 | 0 | 0 | 0 | 0 | 1 | 6 |
| Serranidae | 0 | 1 | 8 | 0 | 2 | 0 | 1 | 1 | 0 | 0 | 2 | 15 |
| Sphyraenidae | 0 | 0 | 1 | 0 | 0 | 0 | 0 | 0 | 1 | 0 | 0 | 2 |
| Stomiidae | 0 | 0 | 2 | 0 | 2 | 0 | 0 | 0 | 0 | 0 | 0 | 4 |
| Syngnathidae | 0 | 0 | 0 | 0 | 0 | 7 | 2 | 0 | 0 | 0 | 0 | 9 |
| Synodontidae | 0 | 0 | 3 | 0 | 4 | 1 | 0 | 0 | 0 | 2 | 0 | 10 |
| Tetraodontidae* | 0 | 0 | 0 | 0 | 0 | 0 | 0 | 2 | 2 | 0 | 0 | 4 |
| Trachipteridae | 0 | 1 | 0 | 0 | 0 | 0 | 0 | 0 | 0 | 0 | 0 | 1 |
| Tripterygiidae* | 0 | 1 | 1 | 0 | 2 | 65 | 3 | 16 | 0 | 2 | 2 | 92 |
| Total identified | 75 | 192 | 689 | 136 | 536 | 286 | 133 | 259 | 198 | 136 | 163 | 2803 |
| Total sampled | 159 | 265 | 791 | 153 | 668 | 405 | 182 | 367 | 282 | 184 | 189 | 3645 |
| # families | 15 | 23 | 38 | 18 | 32 | 19 | 19 | 23 | 23 | 14 | 20 | 62 |
| Fraction identified | 0.47 | 0.72 | 0.87 | 0.89 | 0.80 | 0.71 | 0.73 | 0.71 | 0.70 | 0.74 | 0.86 | 0.77 |
