# Supplementary material for: Coupling and de-coupling of the El Niño Southern Oscillation to the supply of larval fishes to benthic populations in the Hawaiian Islands
Source: PLoS One. 2024 Oct 24;19(10):e0312593. doi: 10.1371/journal.pone.0312593 (PMC11500875; doi:10.1371/journal.pone.0312593)
Supplement: S5 Table — (DOCX) [file pone.0312593.s005.docx]

S5 Table. Estimates of species and family diversity by year based on DNA barcoding and visual identification.

|  |  |  | Bar coding | | | | | Visual identification | | | |
| --- | --- | --- | --- | --- | --- | --- | --- | --- | --- | --- | --- |
| Year | # Tows | Total larvae | Prop. Sequenced | # Species | Prop. ID | # Families | Prop. ID | # Species | Prop. ID | # Families | Prop. ID |
| 2007 | 6 | 159 | 0.64 | 29 | 0.90 | 16 | 0.94 | 7 | 0.17 | 15 | 0.30 |
| 2008 | 9 | 265 | 0.58 | 34 | 0.86 | 22 | 0.97 | 22 | 0.36 | 23 | 0.72 |
| 2009 | 6 | 791 | 0.17 | 60 | 0.82 | 35 | 0.95 | 38 | 0.70 | 38 | 0.87 |
| 2010 | 6 | 153 | 0.43 | 29 | 0.94 | 14 | 0.98 | 24 | 0.34 | 18 | 0.89 |
| 2011 | 9 | 668 | 0.15 | 34 | 0.83 | 21 | 0.90 | 38 | 0.11 | 32 | 0.80 |
| 2012 | 12 | 405 | 0.46 | 38 | 0.81 | 16 | 0.83 | 18 | 0.26 | 19 | 0.71 |
| 2013 | 15 | 182 | 0.17 | 13 | 0.87 | 10 | 0.94 | 23 | 0.31 | 19 | 0.71 |
| 2014 | 15 | 367 | 0.19 | 26 | 0.93 | 16 | 0.97 | 25 | 0.28 | 23 | 0.70 |
| 2015 | 6 | 282 | 0.33 | 27 | 0.89 | 15 | 0.95 | 25 | 0.08 | 23 | 0.74 |
| 2016 | 6 | 184 | 0.12 | 8 | 0.86 | 7 | 0.86 | 11 | 0.43 | 14 | 0.83 |
| 2017 | 6 | 189 | 0.41 | 22 | 0.87 | 18 | 0.99 | 22 | 0.36 | 20 | 0.69 |
| max | 15 | 791 | 0.64 | 60 | 0.94 | 35 | 0.99 | 38 | 0.70 | 38 | 0.89 |
| min | 6 | 153 | 0.12 | 8 | 0.81 | 7 | 0.83 | 7 | 0.08 | 14 | 0.3 |
| median | 6 | 265 | 0.33 | 29 | 0.87 | 16 | 0.95 | 23 | 0.31 | 20 | 0.72 |
